# Metabolomic and kinetic investigations on the electricity‐aided production of butanol by *Clostridium pasteurianum* strains

**DOI:** 10.1002/elsc.202000035

**Published:** 2020-12-06

**Authors:** Philipp Arbter, Wael Sabra, Tyll Utesch, Yaeseong Hong, An‐Ping Zeng

**Affiliations:** ^1^ Institute of Bioprocess and Biosystems Engineering Hamburg University of Technology Hamburg Germany

**Keywords:** BES, butanol production, *Clostridium pasteurianum*, electro‐fermentation, redox metabolism

## Abstract

In this contribution, we studied the effect of electro‐fermentation on the butanol production of *Clostridium pasteurianum* strains by a targeted metabolomics approach. Two strains were examined: an electrocompetent wild type strain (R525) and a mutant strain (dhaB mutant) lacking formation of 1,3‐propanediol (PDO). The dhaB‐negative strain was able to grow on glycerol without formation of PDO, but displayed a high initial intracellular NADH/NAD ratio which was lowered subsequently by upregulation of the butanol production pathway. Both strains showed a 3–5 fold increase of the intracellular NADH/NAD ratio when exposed to cathodic current in a bioelectrochemical system (BES). This drove an activation of the butanol pathway and resulted in a higher molar butanol to PDO ratio for the R525 strain. Nonetheless, macroscopic electron balances suggest that no significant amount of electrons derived from the BES was harvested by the cells. Overall, this work points out that electro‐fermentation can be used to trigger metabolic pathways and improve product formation, even when the used microbe cannot be considered electroactive. Accordingly, further studies are required to unveil the underlying (regulatory) mechanisms.

**Abbreviations**
AEC: Adenylate energy charge
ATP: Adenosine triphosphate
BES: Bioelectrochemical system
NAD: Nicotinamide adenine dinucleotide (oxidized)
NADH: Nicotinamide adenine dinucleotide (reduced)
PDO: 1,3‐propanediol
Rex: Redox sensitive regulator

## 1 INTRODUCTION

**FIGURE 1:**
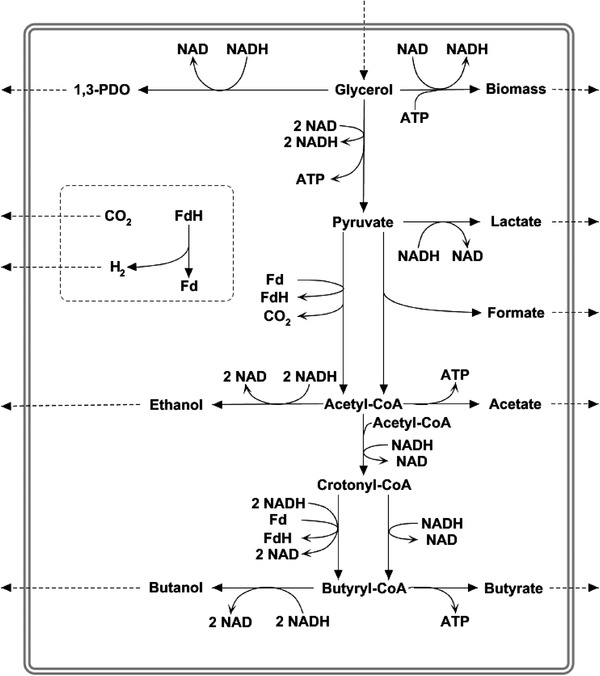
Main metabolic pathways of glycerol fermentation in *C. pasteurianum*. 1,3‐PDO, 1,3‐propanediol; Fd/FdH, oxidized/reduced ferredoxin

The obligate anaerobe bacterium *Clostridium pasteurianum* offers the promising capability to utilize glycerol and convert it into the valuable products 1,3‐propanediol (PDO) and n‐butanol [1, 2, 3], as shown in Figure 1. Glycerol, derived from biodiesel production or biorefineries, is considered to be an appealing feedstock for industrial biotechnology. It can be utilized by various microorganisms and serve as a substrate for the production of other different compounds, such as dihydroxyacetone [4], succinic acid [5], ethanol [6] and microbial oils [7].

Recently, significant progress has been made in improving metabolic engineering tools for *C. pasteurianum* [8, 9, 10, 11]. In this context, Schmitz et al. [10] and Schwartz et al. [11] followed a similar approach to improve butanol production by silencing the conversion of glycerol into PDO: both targeted the first step of PDO synthesis, namely the conversion of glycerol into 3‐hydroxypropanal by the enzyme glycerol dehydratase, which is encoded by the *dhaBCE* genes in *C. pasteurianum*. While Schmitz et al. deleted only the gene for the subunit (*dhaB*), Schwartz et al. knocked out all genes. Both options resulted in the cessation of PDO production, but Schwartz et al. reported that the modified strain could only grow redox balanced in complex media with glycerol as the sole carbon source. Schmitz et al. instead reported successful growth of the mutant in semisynthetic medium on glycerol, demonstrating that redox homeostasis can be maintained solely by the production of butanol. Hence, one goal of this work is to elucidate the redox metabolism of the mutant strain in comparison to the electrocompetent wild type strain (named R525 in the following) of *C. pasteurianum* and quantitatively describe how intracellular cofactor and metabolite levels are altered. For this, fed‐batch cultivations were conducted in a system that allows automated fast‐filtration for the superior quantification of intracellular metabolites [12, 13].

Furthermore, it was reported that *C. pasteurianum* [14, 15] and also other *Clostridia* [16, 17] show improved butanol yield and metabolic shifts by the supply of a cathodic current in mediatorless bioelectrochemical systems (BES). Accordingly, Choi et al. [18] demonstrated that cellular NADH/NAD ratios in *C. pasteurianum* are more than fivefold increased in a cathodic BES, concluding that the cells are *electroactive* and able to harvest electrode derived reducing energy. Nevertheless, calculations yield that in the quoted work, electricity can only account for less than 1% of electrons recovered in the final product [19]. This is interesting since it clearly shows that metabolic shifts and intracellular redox state can already be immensely manipulated by only small amounts of transferred electrons. Similar effects have also been observed in further electricity‐aided studies: Speers and colleagues [20] increased glycerol consumption by *Geobacter sulfurreducens* and the bacterium *Clostridium cellobioparum* for ethanol production by anodic electro‐fermentation, which was also used in another study to improve ethanol and acetate production by engineered *E. coli* strains [21]. Cathodic electro‐fermentation was applied to mixed culture and enabled a doubling in PDO yield [22]. In aerobic cultures, cathodic electro‐fermentation led to an improvement of microbial lipid production by the oleaginous yeast *R. toruloides* [23]. In all of the named studies, electric energy could account for a maximum of 5% of recovered electrons in the product of interest. Unfortunately, these indirect effects of electro‐fermentation are so far poorly understood and seldom quantitatively described. Therefore, the second goal of this work is to study the effect of electro‐fermentation on *C. pasteurianum* strains to contribute to a better understanding of electrochemically induced metabolic shifts towards increased butanol production.

**PRACTICAL APPLICATION**

Butanol is a valuable platform chemical and can be technically produced from glycerol fermentation. The latter is, however, often accompanied by the formation of 1,3‐propanediol. In this work we examined a mutant strain of *Clostridium pasteurianum* lacking 1,3‐propanediol formation and especially regarding electro‐fermentation for its potential to improve butanol production. Both approaches show a strong influence on the microbial redox metabolism and alter the butanol yield. However, the results also indicate that further fundamental research on the underlying mechanisms is required to exploit their full potentials. Here, it is of special interest to note that electro‐fermentation can cause significant metabolic shifts, although only small amounts of electrons from the electrode are harvested by the cells. This highlights the potential use of electro‐fermentation as a general tool to be applied to different fermentation processes, even when the used microbe is not electroactive.

## 2 MATERIALS AND METHODS

### 2.1 Microorganism and medium

An electrocompetent strain *C. pasteurianum *R525 and a PDO lacking mutant strain, referred to as *C. pasteurianum* dhaB mutant strain in this work, were used in this study. The generation of both strains from *C. pasteurianum *DSM 525 was described in detail by Schmitz et al. [10]. Two media were used: Reinforced Clostridial Medium (RCM) and a modified medium from Biebl [24] with pure glycerol (99.5% purity). The medium composition, preparation, and strain maintenance were the same as described by Sabra et al. [25]. Initial glycerol concentration for the fed‐batch cultivations was 25 g L^–1^. The feeding solution consisted of Biebl medium with 500 g L^–1^ pure glycerol. The fermentation medium additionally contained 1 g L^–1^ of d‐xylose as an internal standard for calculation of the cell‐specific concentration of intracellular compounds. No metabolization of d‐xylose was observed.

### 2.2 Cultivation systems and conditions

The fermentations were carried out in a controlled glass bioreactor system with a working volume of 3 L (Bioengineering, Switzerland). The reactor was stirred at 300 rpm by a bottom‐driven stirrer. The feeding was started 8 h after inoculation at a rate of 3 g L^–1^ h^–1^ of glycerol and after 20 h reduced to 1 g L^–1^ h^–1^ overnight. The electricity aided‐fermentations were conducted with the help of a recently developed “All‐In‐One” electrode [14]. The electrode was controlled chronopotentiometrically by an Interface 1000 potentiostat (Gamry, USA). The current supply was started together with feeding after 8 h of cultivation and kept at a constant level of –400 mA. Cell voltage was recorded. Gas volume fractions of hydrogen and carbon dioxide were measured by off‐gas analyzers from BlueSens (Germany). The gas volume off‐gas stream was measured by Milligascounters (Dr. Ing. Ritter Apparatebau, Germany). The cultivation system was equipped with redox and pH probes from Mettler‐Toledo (US). Before all fermentations, the probes were calibrated according to the manufacturer instructions. pH was maintained at 6.0 by the automated addition of 5 mol L^–1^ KOH. The temperature was controlled at 35°C. All reactors and periphery were sterilized for 20 min at 121°C. To establish anaerobic conditions after sterilization, reactors were purged with pure nitrogen for 10 min at 90°C and while cooling down to 35°C. The reactor system for the fed‐batch fermentations was coupled to a self‐developed automated fast‐filtration system.

### 2.3 Fast‐filtration and extraction of intracellular metabolites

For quantification of intracellular metabolites from fast‐sampling, only minor changes were made to the set‐up described by da Luz et al. [13]: the pressure‐driven filtration used nitrogen at 1.5 bar instead of air and the solution for filter washing contained ammonium bicarbonate instead of sodium chloride, since washing the filter with sodium chloride resulted in a non MS compatible sample matrix. The ionic strength of the washing solution was adjusted to match the initial ionic strength of the fermentation medium. LN_2_ was used for quenching of the filters. The whole sampling process, including filtration, filter washing and quenching was finished within 6–8 s. For the optimized quantification of nicotine adenine dinucleotide redox cofactors, the extraction solvents and workflow were adapted and modified from Lu et al. [26]. Extractions were carried out in 16 mL Nalgene polycarbonate centrifuge tubes (Thermo Fisher Scientific, Germany) and started directly after the fast sampling. After quenching, the filters were immediately removed from the filter modules and stored in 5 mL –20°C extraction solvent containing acetonitrile:methanol:water in the volume fractions 40:40:20 and 0.1 mol L^–1^ formic acid. For every fast sampling time point, single two round extractions from three separate filtrations were conducted. First, the quenching solutions containing the submerged filters were vortexed for 30 s and tubes flash‐frozen in LN_2_ for 3 min. Then the solutions were allowed to thaw in an ultrasound bath for 2–3 min and vortexed for 30 s again. After this, 435 μL of ice‐cold ammonium bicarbonate (15% w/v) were added and vortexed briefly. Then, the solutions were centrifuged in a Heraeus Biofuge Stratos centrifuge (Thermo Fisher Scientific, Germany) for 15 min at 13,000 rpm and −19°C. The supernatant was removed and temporarily stored at −20°C. After this, 5 mL of extraction solvent were added to the filter pellet and the extraction procedure was repeated. The supernatants of two rounds of extractions were pooled and finally filtered through a 0.2 μm PVDF syringe filter. Extracts and also filtrates were stored at −80°C until all samples were taken and extracted. Finally, the extracts were lyophilized and stored for further analysis at −80°C.

### 2.4 Quantification of intracellular metabolites

#### 2.4.1 Liquid chromatography‐tandem mass spectrometry (LC‐MS/MS)

Concentrations of pyruvate, acetyl‐CoA, crotonyl‐CoA, butyryl‐CoA, AMP, ADP, ATP, and NAD^+^ were determined by liquid chromatography coupled to tandem mass spectrometry (LC‐MS/MS). Standards of these compounds were purchased in the purest quality available from Sigma‐Aldrich (US). For LC‐MS/MS analysis, lyophilized samples were resuspended in ultrapure water and analyzed by an Agilent Sciex QTRAP 5500 coupled to an Agilent Model 1260 HPLC system (Thermo Fisher Scientific, Germany). The HPLC system was equipped with a Bridge Amide column (3.5 μm, 100 mm × 3 mm) from the company Waters (USA). The pump rate was 350 μL min^–1^. Two eluents were used: water with 20 mM ammonium hydroxide plus 20 mM ammonium acetate (eluent A) and acetonitrile (eluent B). The gradient of the mobile phase was changed in 5 steps over the data acquisition phase of 25 min (Supplementary Table S1). The injection volume was 5 μL and the column temperature 30°C.

#### 2.4.2 Enzymatic quantifications

Total amounts of NAD^+^ plus NADH were quantified by a commercially available enzymatic kit in 96‐well plates. The used kit is named “Amplite™ Colorimetric NAD/NADH Ratio Assay Kit” and was purchased from biomol (Germany). It was used according to the manufacturer's instructions. NADH concentrations were obtained by subtracting NAD^+^ amounts from the measured overall amount of NADH plus NAD^+^.

### 2.5 Quantification of extracellular compounds

#### 2.5.1 High pressure liquid chromatography (HPLC)

Glycerol, PDO, lactate, formate, acetate, ethanol, butyrate, pyruvate, succinate and butanol were quantified by HPLC. For this, cells were centrifuged at 13,000 rpm for 5 min and the supernatant filtered through a 0.2 μm PVDF filter. The HPLC system and method were the same as described by Sabra et al. [27].

#### 2.5.2 Enzymatic quantifications

D‐xylose was quantified by the commercial “D‐Xylose Assay Kit” from the company Megazyme (Ireland) according to the manufacturer's instruction. Concentrations of d‐xlyose from fermenter samples, resuspended filters and filtrates allowed the calculation of the exact filtered sample volume from the automated fast‐filtration. The calculated sample volume (between 600 and 900 μL) was then used for the calculation of the cell‐specific intracellular concentrations.

#### 2.5.3 Determination of cell growth and cell dry weight

Cell growth was tracked by measuring the OD_600_ and correlated with bio dry mass by multiplying the OD values with the constant factor 0.337, according to Groeger et al. [28].

### 2.6 Calculations

#### 2.6.1 Carbon, NADH, and electron recoveries

Molar concentrations (*c*_i_) obtained from the quantification of extracellular metabolites were used for the calculation of carbon and reducing equivalent balances. This was done to check the consistency of the generated data, and to obtain information on the question if electricity derived electrons are harvested by the cells. Carbon (*C*_R_) and NADH (*NADH*_R_) recoveries were calculated according to the following equations [14]:

(1) $$C_{R}=\;\frac{3\;c_{\mathrm{PDO}}+2\;c_{\mathrm{Ethanol}}+4\;c_{\mathrm{Butanol}}+4\;c_{\mathrm{Butyrate}}+\;2\;c_{\mathrm{Acetate}}+\;c_{\mathrm{Formate}}+\;3\;c_{\mathrm{Lactate}}+\;4\;c_{BM}+\;c_{CO_{2}}}{3\;\Delta c_{\mathrm{Glycerol}}}$$

(2) $${\text{NADH}}_{R}=\;\frac{c_{PDO}+\Delta H_{2}}{2\;c_{\mathrm{Acetate}}+c_{\mathrm{Lactate}}+2\;c_{\mathrm{Butyrate}}+c_{BM-H}}$$

where *c*_BM_ refers to the molar concentration of biomass (M = 101 g mol^–1^ at a composition of C_4_H_7_O_2_N), *Δc*_Glycerol_ is the total amount of consumed glycerol divided by the reactor volume, *c*_CO2_ the total measured amount of produced carbon dioxide divided by the reactor volume, *c*_BM‐H_ the amount of formed NADH associated with biomass formation from glycerol (= 13.2 mmol g^–1^), and *ΔH_2_* describes the net amount of NADH oxidized by the bifurcating reaction from crotonyl‐CoA to butyryl‐CoA (Figure 1). This value can be calculated using the following expression:

(3) $$\Delta \;H_{2}=\;c_{H_{2}}-\;c_{H_{2,\mathrm{electrode}}}-c_{H_{2,PFOR}}$$

where *c*_H2_ is the measured total amount of hydrogen divided by the reactor volume, *c*_H2,electrode_ is the volumetric amount of abiotically produced hydrogen by the electrode and *c*_H2,PFOR_ the amount of hydrogen, that is theoretically released by the pyruvate‐ferredoxin‐oxidoreductase. The last term can be calculated from the following relationships:

(4) $$\begin{array}{ccc} c_{H_{2,\mathrm{PFOR}}} & = & c_{\mathrm{Ethanol}}+2\;c_{\mathrm{Butanol}}+2\;c_{\mathrm{Butyrate}}\\ & & +\;c_{\mathrm{Acetate}}-c_{\mathrm{Formate}} \end{array}$$

For the used electrode set‐up, the same reaction medium and conditions, a faradaic efficiency of 81.65% was reported for hydrogen production [14], equaling a H_2_‐production rate of 0.089 mmol min^–1^ at a current of −400 mA. Hence, the amount of abiotically produced H_2_ can be obtained using the following relationship:

(5) $$c_{H_{2,electrode}}=\frac{0.089\;mmol\;\min^{-1}\;\cdot \;t\;}{V_{R}}\;$$

where *t* stands for the total running time of the electrode in min and *V_R_* for the reactor working volume in L. Reducing equivalent balances were calculated on the macroscopic level (*R*_H_^MAKRO^) and by a semi‐pathway approach (*R*_H_^SPATH^) as suggested by Zeng [29]. The latter approach is additionally used in this work since it has been proven to be more sensitive towards measurement errors in comparison to the classical macroscopic electron balances. The reducing equivalent balances were calculated according to the following equations:

(6) $$\;R_{H}^{MAKRO}=\;\frac{16\;c_{PDO}+12\;c_{\mathrm{Ethanol}}+24\;c_{\mathrm{Butanol}}+20\;c_{\mathrm{Butyrate}}+\;8\;c_{\mathrm{Acetate}}+\;2\;c_{\mathrm{Formate}}+\;12\;c_{\mathrm{Lactate}}+\;16\;c_{BM}+\;2\;c_{H2}}{14\;\Delta c_{\mathrm{Glycerol}}}\;$$

(7) $$R_{H}^{SPATH}=\;\frac{c_{\mathrm{Ethanol}}+c_{\mathrm{Butanol}}+4\;c_{\mathrm{Butyrate}}+3\;c_{\mathrm{Acetate}}+c_{\mathrm{Lactate}}+\;4/3\;c_{BM}}{c_{PDO}+c_{\mathrm{Formate}}+c_{H2}}$$

#### 2.6.2 Estimation of cell‐specific rates

The cell‐specific maximal growth rate *μ*_max_ was determined by computing the natural logarithm of the measured values for cell dry weight. Then, linear regression was applied to obtain the slope of the linearized growth curve. The corresponding plots can be found in the supplementary.

The cell‐specific uptake and production rates for glycerol, PDO and butanol were obtained in a similar approach as used by Niklas et al. [30]: cubic hermite splines were fitted to the measured extracellular metabolite (*c*_i_) and biomass (*c*_x_) data and then the specific rates (*q*_i_) were obtained by the following equation:

(8) $$q_{i}=\;\frac{dc_{i}/dt}{c_{x}}$$

Fitting was conducted in Matlab (Version R2013a) using the function *pchip*. Spline first derivatives were obtained by the function *fnder*.

#### 2.6.3 Adenylate energy charge

The adenylate energy charge (AEC) was obtained from the measured intracellular molar concentrations of AMP (*c*_AMP_), ADP (*c*_ADP_) and ATP (*c*_ATP_) by the following equation [31]:

(9) $$AEC\;=\frac{c_{ATP}+\;0.5\;c_{ADP}}{c_{ATP}+c_{ADP}+c_{AMP}}\;$$

## 3 RESULTS AND DISCUSSION

**FIGURE 2:**
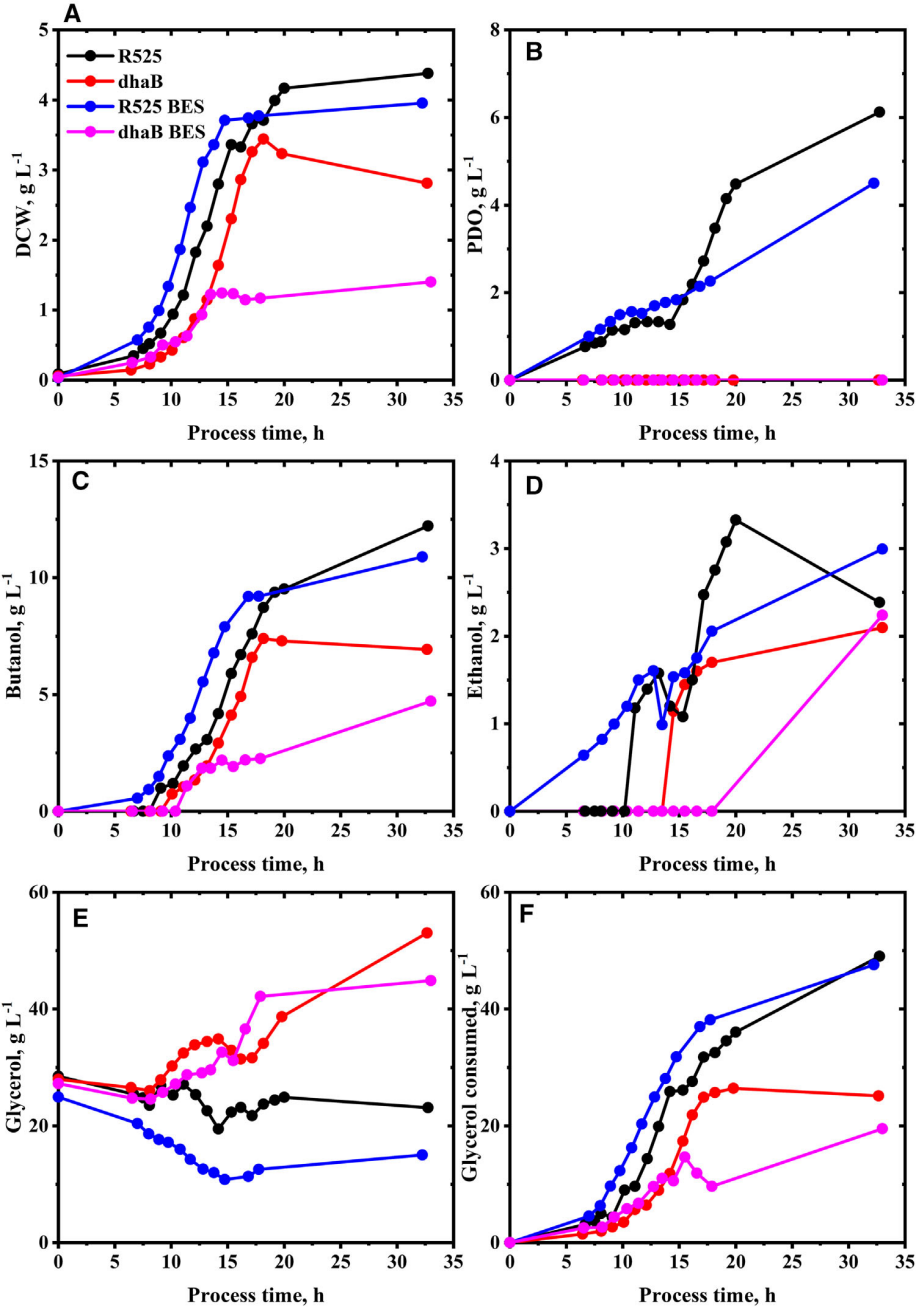
Measured dry cell weight (DCW) (A), extracellular concentrations of 1,3‐propanediol (PDO) (B), butanol (C), ethanol (D), glycerol (E) and total amount of consumed glycerol (F) during the fed‐batch cultivation of *C. pasteurianum* in Biebl medium. Initial glycerol concentration was 25 g L^–1^. Feed started after 8 h with 3 g L h^–1^, reduced after 20 h to 1 g L h^–1^. Application of −400 mA current after 8 h in the BES cultivations

**FIGURE 3:**
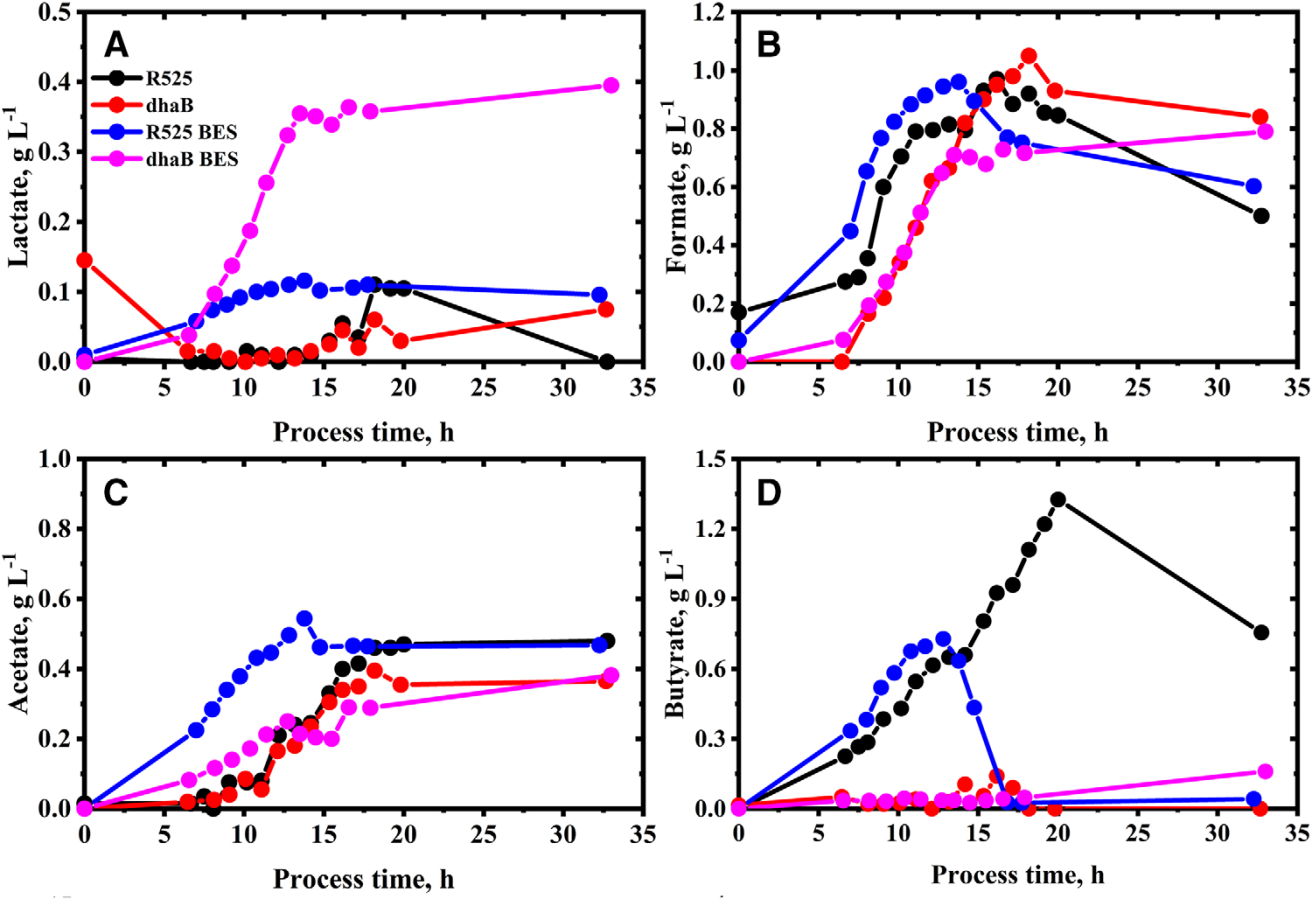
Extracellular concentrations of lactate (A), formate (B), acetate (C), and butyrate (D) during the fed‐batch cultivation of *C. pasteurianum* in Biebl medium. Initial glycerol concentration was 25 g L^–1^. Feed start after 8 h with 3 g L h^–1^, reduced after 20 h to 1 g L h^–1^. Application of −400 mA current after 8 h in the BES cultivations

**FIGURE 4:**
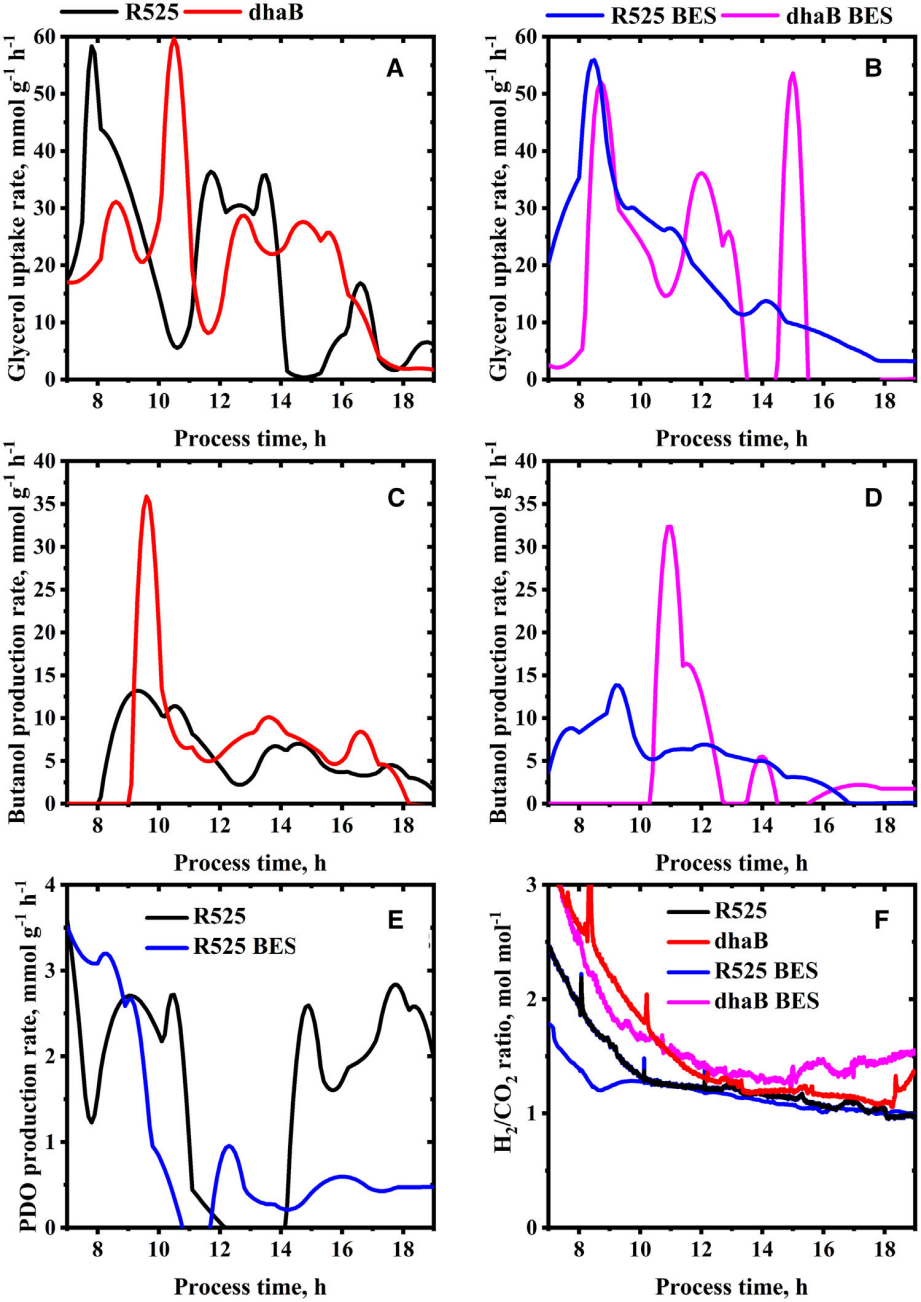
Estimated cell‐specific rate of glycerol uptake (A, B) and butanol production (C, D) of *C. pasteurianum* cells during fed‐batch and electricity‐aided fed‐batch cultivation. PDO production is only shown for the R525 strain (E), since no PDO production was observed for the dhaB mutant strain. F: online measured H_2_/CO_2_ ratio from all four conducted cultivations

**FIGURE 5:**
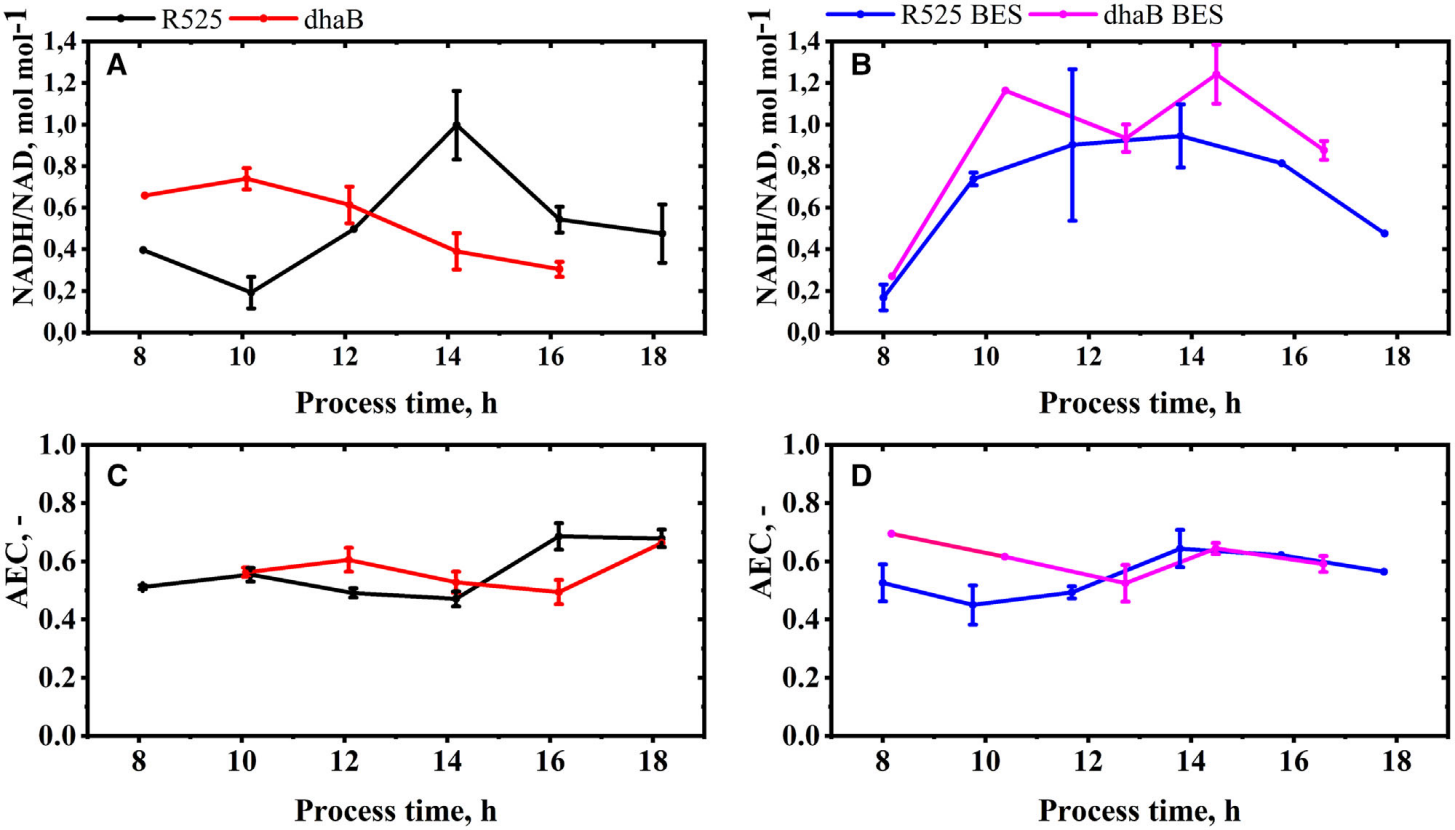
Intracellular NADH/NAD ratio (A, B) and adenylate energy charge (C, D) of *C. pasteurianum* cells during fed‐batch and electricity‐aided fed‐batch cultivation. Errors indicate the standard deviation of concentrations obtained from three separate metabolite extractions. When no error bar is stated, the average of two samples is shown

**FIGURE 6:**
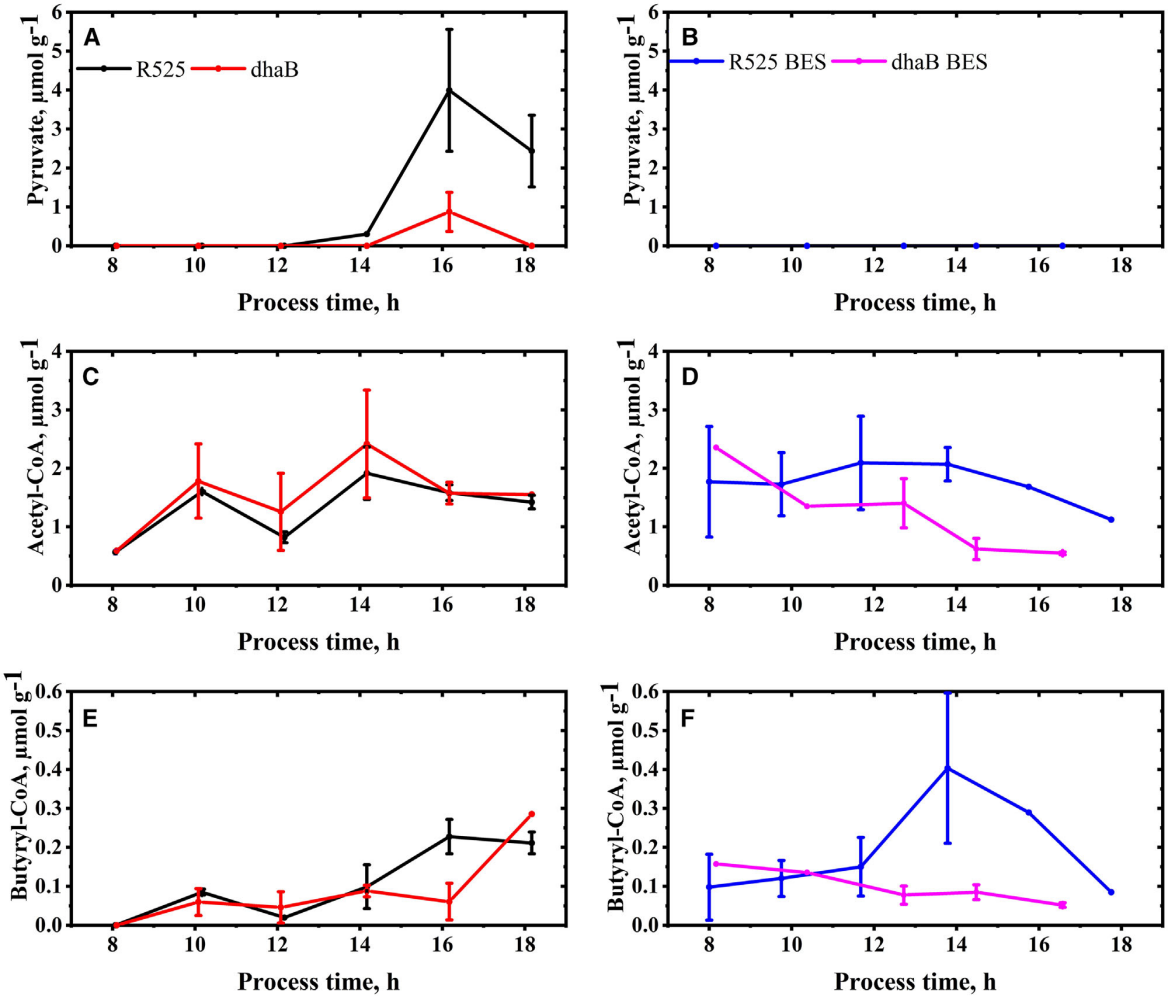
Intracellular concentrations of pyruvate (A, B), acetyl‐CoA (C, D), and butyryl‐CoA (E, F) in *C. pasteurianum* cells during fed‐batch and electricity‐aided fed‐batch cultivation. Errors indicate the standard deviation of concentrations obtained from three separate metabolite extractions. When no error bar is stated, the average of two samples is shown

In total, four fed‐batch cultivations were performed: two with the R525 strain and two with the dhaB mutant strain. Both strains were tested in standard stirred tank bioreactors and additionally in a BES with an applied cathodic current of −400 mA. For all experiments, extracellular substrate and product concentrations were determined (Figures 2 and 3), from which carbon and reducing energy balances were derived (Table 1). Also, cell‐specific substrate uptake and metabolite production rates were estimated from the data (Figure 4). Due to the used estimation procedure and low sampling intervals, the absolute values of the estimated cell‐specific rates should be regarded with caution, but enable a fair comparison of the cell's metabolic state between the different experiments. The dataset is complemented by intracellular concentrations of targeted metabolites obtained from fast‐filtration sample treatment and quantified in 2 h intervals between 8 and 18 h of the cultivation (Figures 5 and 6). In the following, first, the R525 strain is compared to the dhaB mutant strain without electricity and subsequently, the effect of cultivation in the BES is described and discussed.

**TABLE 1:** Applied current in the BES cultivations (I), maximal specific growth rate (*μ*_max_), carbon recovery (*C*_R_), NADH recovery (NADH_R_), macroscopic reducing energy recovery (*R*_H_^MAKRO^), semi‐pathway reducing energy recovery (*R*_H_^SPATH^), final yield for 1,3‐propanediol (*Y*_PDO/Gly_), final yield for butanol (*Y*_But/Gly_), final 1,3‐propanediol to butanol ratio (*Y*_But/PDO_), final 1,3‐propanediol concentration (*c*_PDO_) and final butanol concentration (*c*_But_) for the conducted fed‐batch cultivations

Strain	I, A	*μ*_max_, h^–1^	*C*_R_, %	NADH_R_, %	*R*_H_^MAKRO^, %	*R*_H_^SPATH^, %	*Y*_PDO/Gly_, mol mol^–1^ (g g^–1^)	*Y*_But/Gly_, mol mol^–1^ (g g^–1^)	*Y*_But/PDO_, mol mol^–1^ (g g^–1^)	*c*_PDO_, mmol L^–1^ (g L^–1^)	*c*_But_, mmol L^–1^ (g L^–1^)
R525	–	0.31	102.8	133.9	103.5	106.2	0.13 ± 0.01 (0.11 ± 0.01)	0.31 ± 0.01 (0.25 ± 0.01)	2.38 (2.32)	80 ± 4 (6.13 ± 0.31)	165 ± 8 (12.22 ± 0.61)
*dhaB* mutant	*–*	0.33	101.6	102.6	102.3	100.5	0.00 ± 0.00 (0.00 ± 0.00)	0.33 ± 0.01 (0.26 ± 0.01)	–	0.00 ± 0.00 (0.00 ± 0.00)	98 ± 5 (7.30 ± 0.36)
R525	−0.4	0.30	99.4	91.7	96.1	103.4	0.09 ± 0.01 (0.07 ± 0.01)	0.29 ± 0.01 (0.23 ± 0.01)	3.22 (3.14)	59 ± 3 (4.50 ± 0.22)	147 ± 7 (10.89 ± 0.54)
*dhaB* mutant	−0.4	0.22	97.1	119.7	99.5	98.5	0.00 ± 0.00 (0.00 ± 0.00)	0.24 ± 0.02 (0.19 ± 0.02)	–	0.00 ± 0.00 (0.00 ± 0.00)	64 ± 3 (4.71 ± 0.24)

### 3.1 Comparison of the R525 and dhaB mutant strains without electricity

Comparing the growth curves of the R525 and dhaB mutant strains, it appears obvious that the dhaB mutant strain offered a slightly longer lag‐phase but reached a higher maximal specific growth rate with 0.33 h^–1^ (0.31 h^–1^ for the R525 strain). Both strains began declining into the stationary phase after about 15 h of cultivation. Growth and gas production for the dhaB mutant strain ceased abruptly after about 18 h. With 2.81 g L^–1^ the mutant strain also yielded a lower final biomass than the R525 strain with 4.38 g L^–1^.

Regarding product formation, the most significant difference between the two strains is that the dhaB strain expectedly produced no PDO. Furthermore, the dhaB strain produced less butyrate than R525. Even though the value of final butanol concentration for the R525 (12.22 g L^–1^) was higher than that for the dhaB mutant strain (7.30 g L^–1^), the mutant reached an overall higher butanol yield than the electrocompetent R525 strain (0.33 mol mol^–1^ vs. 0.31 mol mol^–1^). This is because the R525 strain consumed a much higher total amount of glycerol (49.02 g L^–1^) than the dhaB strain (26.40 g L^–1^). Nevertheless, it appears surprising that the butanol yields display such minor difference since shuffling molecules towards butanol and butyrate production is the only option for the dhaB mutant strain to regenerate excess amounts of NADH by exploitation of the electron bifurcating reaction of crotonyl‐CoA to butyryl‐CoA [32]. Here, the data suggest that in total, the dhaB mutant strain shuffled 57% of the flux from crotonyl‐CoA to butyryl‐CoA through this reaction while for the R525 strain, it were only 24%. The higher use of the bifurcation pathway by the dhaB mutant can also be recognized by the higher H_2_/CO_2_ ratio (Figure 4F). In contrast to the dhaB strain, the R525 strain also has the option to produce PDO as a sink for NADH. Overall, the R525 regenerated 45% of the produced NADH through this pathway. A further difference in product formation can be recognized via evaluation of the final distributions for reducing energy and carbon (Supplementary Table S2). Here, one can see that the dhaB strain shuffled more carbon atoms and reducing energy towards ethanol. However, ethanol production in the dhaB strain was first triggered at later fermentation time points, when growth slowed down or ceased already, allowing the strain to maintain NADH neutral glycerol consumption.

The different mechanisms of the two strains to maintain redox homeostasis is also reflected in the development of the intracellular NADH/NAD ratios, as shown in Figure 5. The absolute quantified cell‐specific concentrations of intracellular NAD and NADH can be found in the supplementary. At the time points of feed start and first fast‐filtration after 8 h, initial NADH/NAD ratio in the dhaB mutant strain (0.66) was higher than that in the R525 strain (0.44). At this time point of cultivation, the R525 strain did already produce PDO (as reflected in Figure 4E), allowing to maintain a lower NADH/NAD ratio than the dhaB mutant strain, which lacks this option. In agreement with the observations of Johnson and Rehmann [33], PDO formation in *C. pasteurianum* generally begins prior to butanol production and potentially served the R525 strain as an initial valve for excess intracellular reducing energy caused by initially high substrate uptake rates. The dhaB mutant instead solely relies on butanol production, which began later for both strains after about 10 h. In this context, it is notable that the estimated rate for butanol production at this time point is more than double as high for the dhaB mutant strain as for the R525 strain. From approximate 11 h onwards, the butanol production by the dhaB strain was then maintained in a magnitude of 5 to 10 mmol g^–1^ h^–1^ until 17 h and decreased to zero when growth and substrate uptake ceased. In the dhaB mutant strain, the production of butanol between 10 to 18 h was evidentially sufficient to drive a constant lowering of the observed intracellular NADH/NAD ratio in this period. After 18 h no NADH could be detected in the samples for the dhaB strain. Therefore, no NADH/NAD ratio is displayed. In contrast to the dhaB strain, the NADH/NAD ratio in the R525 strain was increasing between 10 to 14 h, although the butanol production rate reached similar levels as for the dhaB strain. As outlined earlier, this might be because the strain did not use the bifurcation pathway to the same extent as the dhaB mutant. Interestingly, the R525 strain appeared to tackle the high NADH/NAD ratio after 14 h by reactivating PDO production and also increasing ethanol levels. Also, the substrate uptake rate declined after 14 h and began increasing when the NADH/NAD ratio in the R525 strain was at a lower level again.

Conclusively based on the presented results, the following summarized mechanism is suggested for the dhaB mutant strain's redox metabolism: initially high substrate uptake rates and PDO deficiency lead to elevated NADH/NAD ratios. Compared to the R525 strain, this results in a higher upregulation of the butanol producing pathway and allows sufficient and constant NADH reoxidation by the activated bifurcating reaction of crotonyl‐CoA to butyryl‐CoA. In *C. acetobutylicum*, butanol formation is controlled by the redox sensitive regulator *Rex*, which is activated (derepressed) by high NADH/NAD ratios and governs the expression of genes involved in butanol synthesis and NADH consuming pathways [34, 35]. The *Rex* protein of *C. pasteurianum* possesses 76% identity to the corresponding protein from *C. acetobutylicum* [34]. Hence, it is assumed that the observed increase in the butanol production for the dhaB mutant strain is driven by *Rex* activation at high NADH/NAD ratios. The assumption might also be supported by the fact that compared to the R525 strain, less butyrate was produced. In *C. acetobutylicum*, *Rex* has also been shown to cause downregulation of the two enzymes phosphate butyryltransferase and butyrate kinase [36]. Both are inevitably required for butyrate formation. For the R525 strain, butyrate production took place from the beginning of fermentation. The dhaB strain contrarily only showed significant butyrate production after about 13 h at already decreased NADH levels and also lowered butanol production rates. Schwartz et al. [11] also observed lower butyrate production and re‐utilization in *Rex* mutants of *C. pasteurianum*. When butyrate is re‐assimilated *C. pasteurianum*, the butyrate molecule reacts with acetoacetyl‐CoA and butyryl‐CoA plus acetoacetate are formed [1]. The latter is then converted into acetone. However, no acetone could be detected in the samples by HPLC, even when butyrate was taken up by the cells.

Also, while determined intracellular concentrations for acetyl‐CoA and butyryl‐CoA followed a similar pattern and lie within the same range in both strains during the tracked period from 8 to 18 h (Figure 6), a further indicator for activation of *Rex* genes is the lower intracellular concentration of pyruvate in the dhaB mutant strain. Except for the data from 16 h, pyruvate was always under the detection limit of the applied quantification method in the dhaB samples. This points towards a higher flux downstream of pyruvate compared to the R525 strain. Crotonyl‐CoA could not be detected in any of the extracted samples. Although the hypothesized mechanism of *Rex* activation in the dhaB mutant strain might explain why the strain's NADH/NAD ratio was decreased again without PDO formation, it remains unclear why the strain abruptly stopped growing after 18 h. One possible explanation might be that the initially high NADH/NAD ratio somehow led to overcompensation of NADH reoxidation by *Rex* controlled pathways, which then decreased absolute NADH levels in the cells below a critical value for cell viability. A similar phenomenon was observed for *Streptococcus mutants* strains [37], but without resulting in cell death. Hypothetically, this overshooting of *Rex* associated pathways did not take place in the R525 strain since PDO formation allows fast intracellular correction of critically high NADH levels and less *Rex* regulated proteins are transcribed. This also highlights the importance of quantifying not only the often stated NADH/NAD ratios in metabolic investigations but also the absolute intracellular concentrations of the nicotinamide adenine dinucleotides (see supplementary). Nonetheless, in summary, the data of the fed‐batch cultivations conducted without electricity clearly show that *C. pasteurianum* can grow redox balanced in semisynthetic medium even without producing PDO. In the following experiments, it was investigated which effect the supply of cathodic current has on the two strains.

### 3.2 Influence of cathodic current on the two strains

Compared to the fermentations conducted without electricity, the R525 strain showed similar growth behavior with electricity. On the other hand, the growth curve of the dhaB mutant strain delivers a different picture: with the application of −400 mA, the strain reached a 33% lower maximal specific growth rate with 0.22 h^–1^. Moreover, cell growth stopped after 14 h and the final biomass value was the lowest of all conducted fermentations with only 1.40 g L^–1^.

The macroscopic electron balances (Table 1) suggest that the strains were not able to harvest a surplus of electricity derived electrons and incorporate them into the measured products. However, the application of electric current showed an influence on product formation, e.g. the molar ratio of produced butanol to PDO is increased for the R525 strain from 2.38 to 3.22. Since the molar yield for butanol was not higher for both strains in the BES, this was achieved by the R525 strain by shuffling less substrate towards PDO, resulting in a lower yield for PDO compared to the fermentations without artificial electron supply. A further electricity induced change in the product formation can be found in the butyrate concentration: in contrast to the fermentation without electricity, R525 stopped producing butyrate after 15 h. In fact, the butyrate concentration even decreases to almost zero until the end of fermentation. The decreased butyrate production in the BES cultivations is in agreement with the observation of Engel et al. [16], who reported 57% lower butyrate yield in an optimized BES for the cultivation of *C. acetobutylicum*. Again, no acetone was detected when butyrate was presumably re‐assimilated by the cells. Moreover, the R525 and also the dhaB mutant strain produced more lactate when supplied with electricity.

Even though Choi et al. [18] used glucose as substrate (and not glycerol), used graphite felt electrodes (and not platinized titan), and worked chronoamperometrically at much lower voltages, similar observations were made in this work regarding the NADH/NAD ratio when the *C. pasteurianum* cells were exposed to an artificial electron source: within 2 h after starting current flow ratios increased from 0.17 to 0.74 (R525) and from 0.27 to 1.16 (dhaB mutant). The lower increase in the R525 strain can be accounted for the production of PDO, which again was observed before butanol production. The dhaB mutant is not able to produce PDO and the NADH/NAD ratio only slightly began to decrease when butanol production rate peaked after about 11 h. Similar as for the fed‐batch fermentations without electricity, the specific butanol production of the dhaB mutant strain was almost doubled compared to the R525 strain. For both BES fed‐batch cultivations, the intracellular NADH/NAD ratio remained >0.8 until 16 h of cultivation. Both options, butanol and PDO production, were not sufficient in the BES scenario to lower the ratio into regions that were observed in the non‐BES cultivations. Only for the R525 strain, the NADH/NAD ratio was decreased again after 18 h in the BES. In the BES, the dhaB mutant strain's maximal NADH/NAD ratio was also absolutely higher than in the cultivations without electricity. In this case, it is assumed that the high cofactor ratio (1.16 after about 10 h) and insufficient NADH reoxidation limited cell growth, as also observed for other *Clostridia* at excess NADH/NAD ratios [38].

It is assumed that the increase of intracellular NADH/NAD levels is mainly driven by the electrochemically caused reduction of the fermentation broth, as also reflected in the measured online values of the redox potential (ORP). ORP is well‐known to influence gene expression [39, 40] and metabolic regulation on different levels for several microorganisms [41, 42, 43]. The monitored ORP online values are supplemented (Figure S4). In BES cultivations, the ORP was 50–100 mV lower than in fermentations without the electrode. It is expected that the lower ORP value in the BES are due to the additionally electrochemically generated amounts of hydrogen or the electrochemical reduction of excreted cellular redox‐active compounds. Also, it was shown in other bioelectrochemical studies with *C. pasteurianum* that iron compounds have an influence on ORP [14, 15]. Usually, the ORP values for iron‐limited cultivations are more negative. Accordingly, the use of electricity might impose electrochemical adsorption of iron compounds and lead to a decrease of iron species in the medium, as demonstrated for H‐cell reactors by Utesch and Zeng [14]. The described mechanism can then also contribute to the ORP decrease of the fermentation broth. Furthermore, it is well‐known that iron availability has a significant effect on product formation and distribution in *C. pasteurianum* [44, 45]. It cannot be excluded that the electrochemically imposed change of iron availability in the medium also contributed to the observed metabolic changes and the shift in product pattern. Nonetheless, in the referred work of Groeger et al., iron limitation led to a decrease of the butanol to PDO ratio, which is contrary to the results of this study.

Based on the high NADH/NAD ratios in the BES, it is expected that also for the R525 strain, *Rex* controlled pathways were upregulated in the electricity‐aided cultivation. This might have been especially the case after about 14 h of cultivation when butyrate production ceased. Simultaneously, glycerol uptake trended toward zero and also the PDO production rate did not reach the same level again as in the non‐BES cultivation. This finally led to a lower PDO concentration and a higher butanol to PDO ratio for the R525 strain in the BES. Another hint towards *Rex* influence was the higher lactate production in the BES. The enzyme lactate dehydrogenase is activated at high NADH/NAD ratios upon *Rex* depression, triggering lactate production in *C. acetobutyricum* [34]. Contrarily, Schwartz et al. [11] could not identify *Rex* sequences in the genes of the lactate dehydrogenase of *C. pasteurianum*, but unexpectedly higher lactate production was experimentally observed in cultivations of *Rex* lacking mutant strains.

For all intracellular BES samples, no pyruvate and also no crotonyl‐CoA could be detected in the extracted samples. The concentration course of butyryl‐CoA and acetyl‐CoA also indicate that the BES cultivation shows a strong influence on the metabolic pathways downstream of pyruvate, what was presumably driven by the artificially increased NADH/NAD ratio. But accordingly, the R525 strain did only shuffle 9% of the flux from crotonyl‐CoA to butyryl‐CoA through the bifurcation pathway, equalling the regeneration of only 17% of the total produced NADH. Hence, higher relative usage of the bifurcation pathway for elevated NADH levels was not observed in the R525 strain. The opposite was the case for the dhaB mutant strain: here, the usage of the bifurcation reaction increased from 57% (no BES) to 72% (BES). The differing activities of the bifurcating reaction were also again reflected in the recorded H_2_/CO_2_ ratio: the ratio for the mutant was higher than for the R525 strain in the BES, but the signal from the R525 of the electricity‐aided cultivation was not higher than the signal from the cultivation conducted without the electrode. Summarizing, this suggests that a higher NADH/NAD alters the activity of the butanol and butyrate production pathway but does not necessarily increase the usage of the bifurcation reaction. This again points out that other yet unconsidered or unknown regulations and mechanisms play a role in the BES. In this context, one possible explanation for the early growth cessation of the dhaB strain in the BES might be that the prospectively lower iron availability resulted in a decrease of electron transfer flavoproteins. These proteins are involved in the bifurcation reaction of crotonyl‐CoA to butyryl‐CoA. Groeger et al. [45] showed that these proteins have a 1.5‐1.7 higher abundance at iron excess conditions. Hence, it is hypothesized that a decrease of the iron availability in the BES limited the use of the bifurcation pathway, which is essential for the dhaB mutant to maintain redox homeostasis under growth conditions. Besides, the lower iron availability in the BES might also be one explanation for the fact that the butanol yield from glycerol is lower than that in the non‐BES cultivations, although *Rex* related pathways might be upregulated by the high NADH/NAD ratios.

Concerning the measured AEC (as displayed in Figure 5), it is remarkable that in the BES the dhaB mutant showed higher initial values than all other strains. This might be explained by the assumption that the strain is limited in anabolic pathways due to high initial NADH/NAD ratios. Except for this, the magnitude and also chronological development of the AEC in the other cultivations appeared similar. Here, it is surprising that the dhaB mutant (non‐BES) reached a similar growth rate as the R525 strain, despite the fact that for the dhaB strain only trace amounts of butyrate and similar amounts of acetate were produced.

## 4 CONCLUDING REMARKS

In the present work, the growth and metabolism of *C. pasteurianum* grown on a semisynthetic medium on glycerol without the formation of PDO were elucidated. In this case, cellular redox metabolism is heavily perturbed and initial intracellular NADH/NAD ratios are elevated, but subsequent activation of butanol production enables a sufficient reoxidation of NADH by exploiting the bifurcation reaction of crotonyl‐CoA to butyryl‐CoA. Furthermore, cultivation in a cathodic BES showed that *C. pasteurianum* is not electroactive but that the applied current still causes a momentous increase of the intracellular NADH/NAD ratio. This results in a higher butanol to PDO ratio for the electrocompetent wild‐type strain of *C. pasteurianum*. Without the ability of PDO formation, growth of the PDO lacking dhaB mutant strain ceased early in the BES. Overall, the results suggest that the silencing of the PDO pathway and electro‐fermentation represent promising approaches to improve butanol production by *C. pasteurianum*. Nonetheless, more detailed work in this context is required to understand which particular yet unknown mechanisms lead to the observed elevation of the intracellular NADH/NAD ratio in the BES and if the higher observed activity of the butanol producing pathways can indeed be accounted to the upregulation of *Rex* controlled enzymes. In this context, detailed transcriptomic or proteomic studies might help to unveil these uncertainties. For further BES studies, it would be interesting to elucidate if a flavin based mechanism is responsible for the transfer of small amounts of electrode derived reducing equivalents into the cells, as suggested for *C. acetobutylicum* [46, 47]. Here, it would be of special concern to doubtlessly clarify if almost negligible small (not quantifiable on a macroscopic level) amounts of transferred electrons can indeed facilitate the observed increase in the NADH/NAD ratio and metabolic shifts. This might highlight the use of electro‐fermentation not mainly as an electron source or sink but also as an indirect tool to control microbial metabolism by secondary (regulatory) effects.

## CONFLICT OF INTERESTS

The authors have declared no conflicts of interest.

## Supporting information



Supplementary information

## Data Availability

The data that support the findings of this study are available from the corresponding author upon reasonable request.
