# Supplementary material for: Metabolomic and kinetic investigations on the electricity‐aided production of butanol by Clostridium pasteurianum strains
Source: Eng Life Sci. 2020 Dec 6;21(3-4):181–95. doi: 10.1002/elsc.202000035 (PMC7923553; doi:10.1002/elsc.202000035)
Supplement: Supplementary file 1 — Supplementary information [file ELSC-21-181-s001.pdf]

Supplementary

Metabolomic and kinetic investigations on the electricity-aided production of butanol by *Clostridium pasteurianum* strains

Philipp Arbter[1], Wael Sabra[1], Tyll Utesch[1], Yaeseong Hong[1] and An-Ping Zeng[1]

[1]Institute of Bioprocess and Biosystems Engineering, Hamburg University of Technology, Denickestraße 15, D-21073 Hamburg, Germany

Correspondence: Prof. An-Ping Zeng (aze@tuhh.de), Institute of Bioprocess and Biosystems Engineering, Hamburg University of Technology, Denickestraße 15, D-21073 Hamburg, Germany

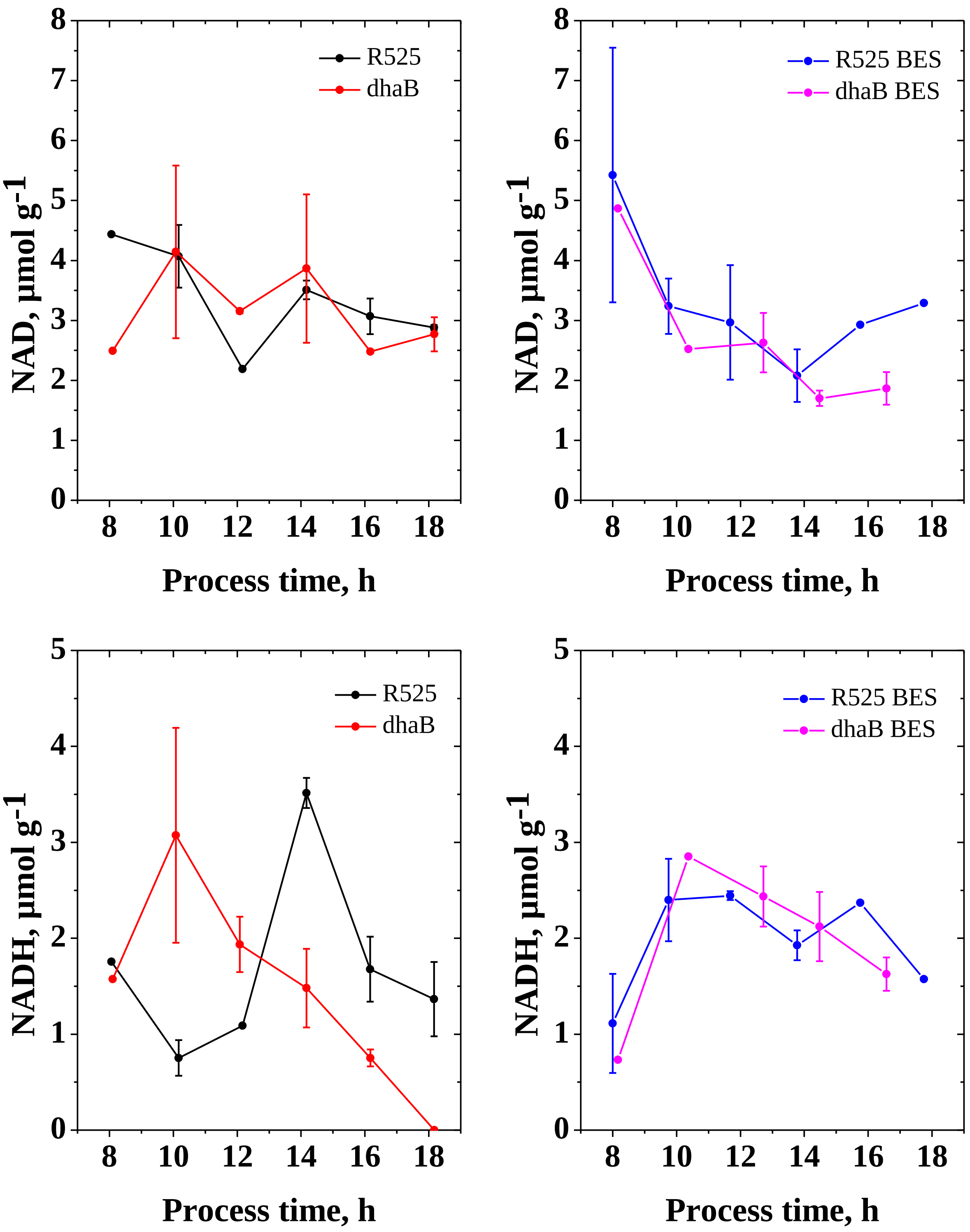

Figure S1: Absolute intracellular concentrations of NAD and NADH of *C. pasteurianum* cells during fed-batch (left column) and electricity-aided fed-batch (right column) cultivation. Errors indicate standard deviation of concentrations obtained from three separate metabolite extractions. When no error bar is stated, the average of two samples is shown. Black: R525 strain; Red: dhaB mutant strain; Blue: R525 strain in BES; Magenta: dhaB mutant strain in BES.

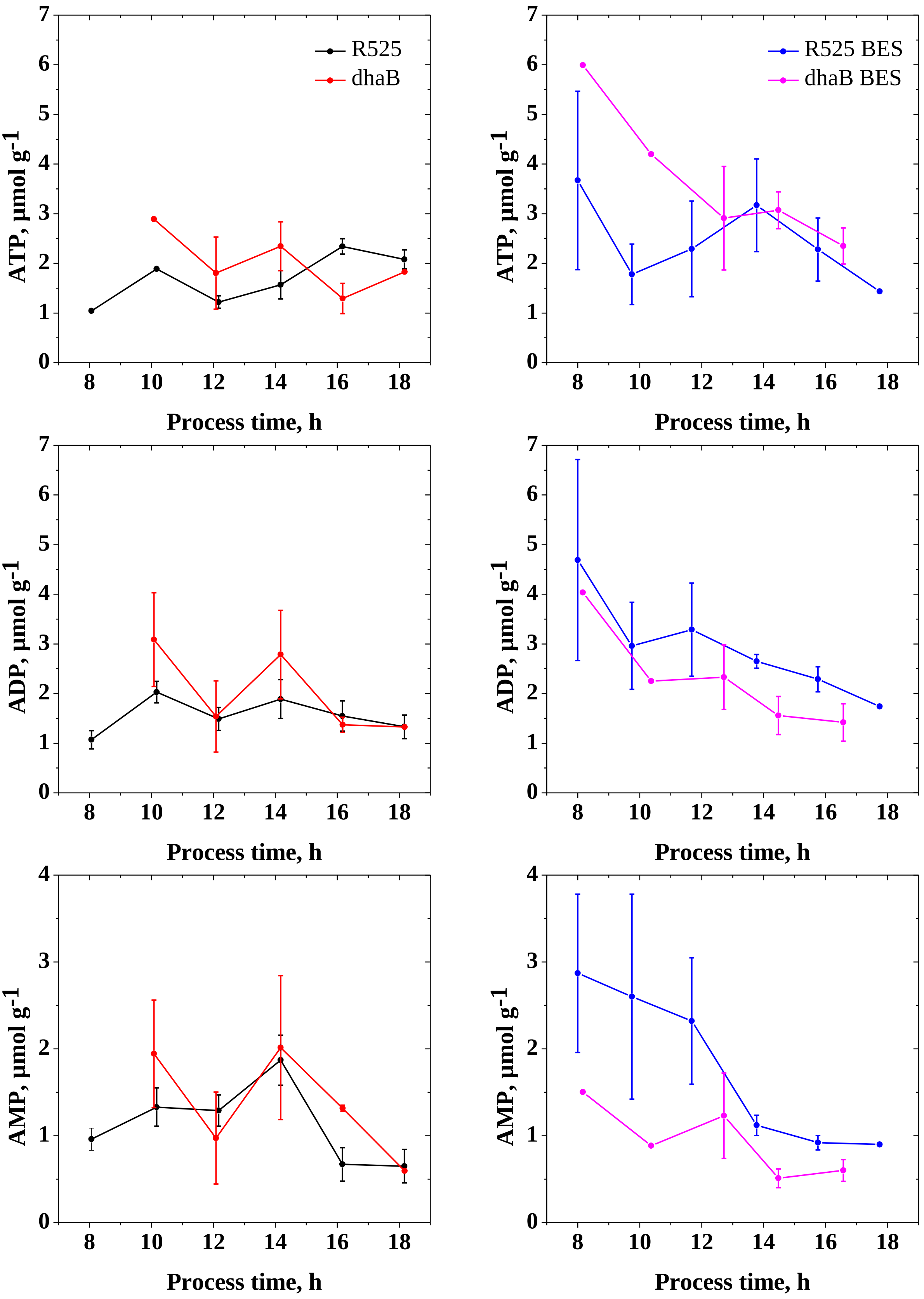

Figure S2: Absolute intracellular concentrations of ATP, ADP and AMP of *C. pasteurianum* cells during fed-batch (left column) and electricity-aided fed-batch (right column) cultivation. Errors indicate standard deviation of concentrations obtained from three separate metabolite extractions. When no error bar is stated, the average of two samples is shown. Black: R525 strain; Red: dhaB mutant strain; Blue: R525 strain in BES; Magenta: dhaB mutant strain in BES.

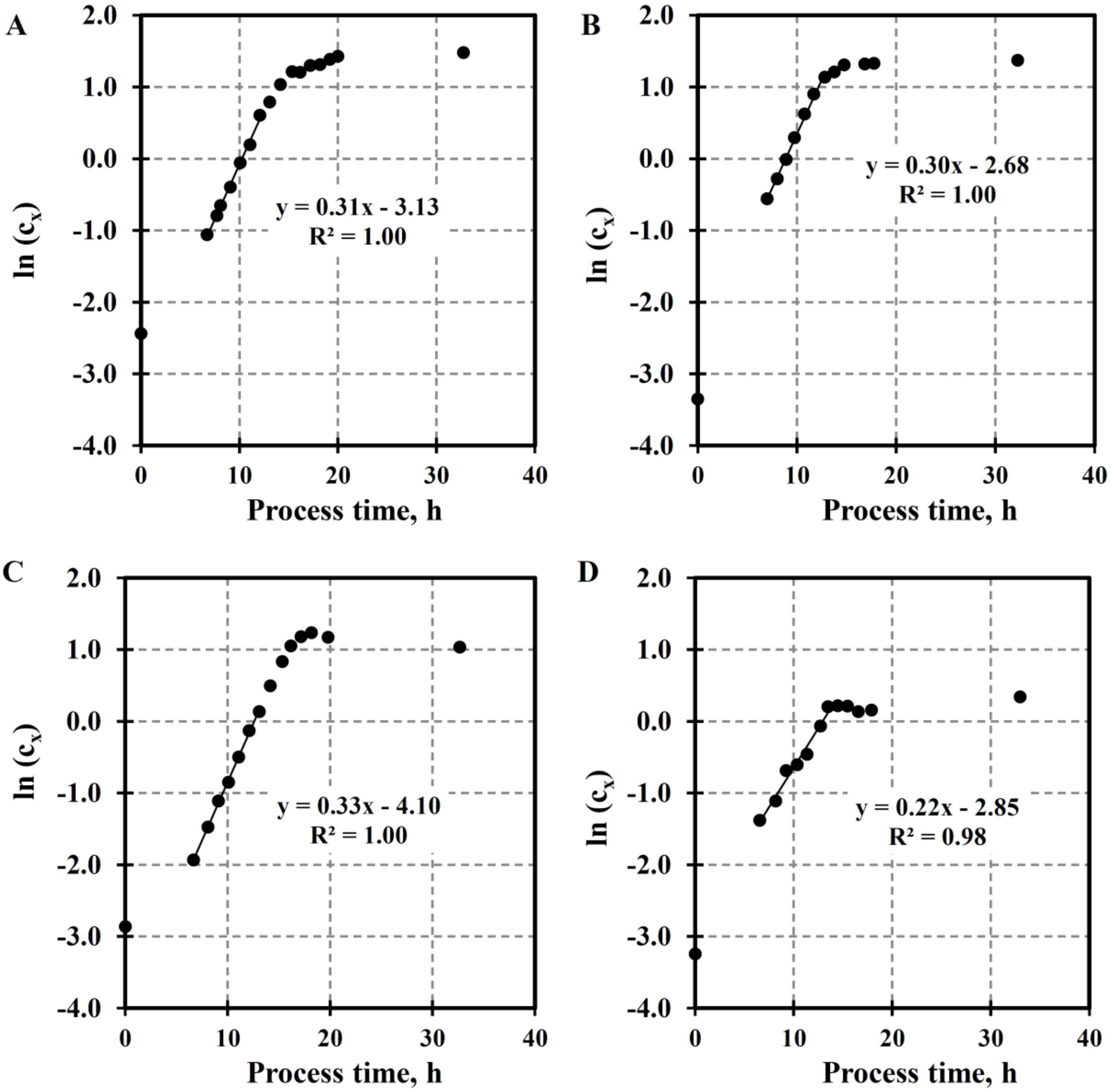

Figure S3: Determination of the cell-specific growth rate μ_{max} (in h^{-1}) by linearization of the experimentally determined cell dry weight and linear regression. A) R525; B) R525 BES; C) dhaB; D) dhaB BES.

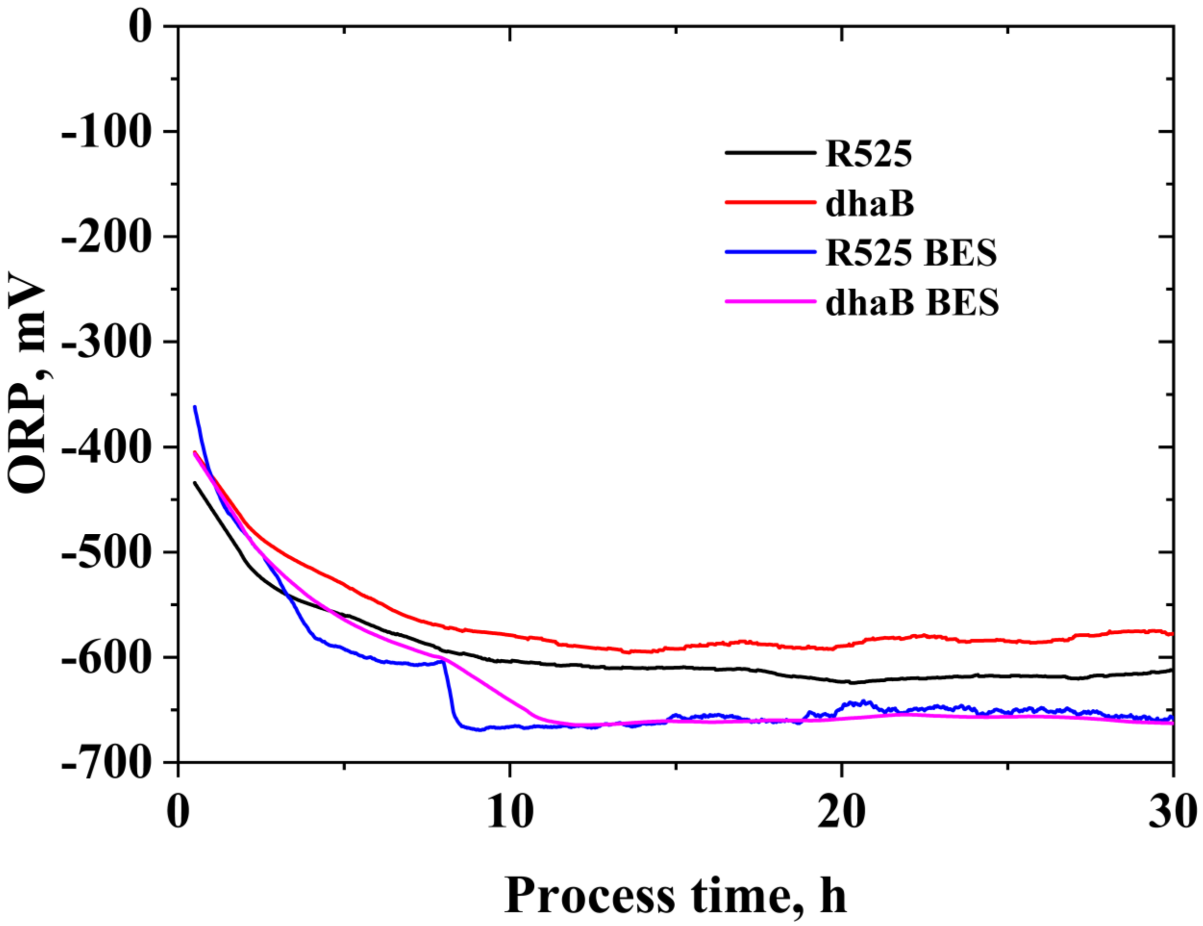

Figure S4: Online measured values of the oxidative-reduction potential (ORP).

Table S1: Mobile phase elution gradient of the LC-MS/MS

Time, min	Eluent A*, %	Eluent B**, %
0	15	85
3	70	30
12	98	2
16	98	2
16,1	15	85
25	15	85

*20 mM NH_4OH and 20 mM CH_3COONH_4 in H_2O

**ACN

Table S2: Final distribution of reducing energy (top) and carbon atoms (bottom) for fed-batch cultivations of *C. pasteurianum* strains. BES indicates application of -0.4 A. PDO = 1,3-propanediol; Eth. = ethanol; But. = butanol; Acet. = acetate; Form. = formate; Suc. = succinate; Lac. = lactate; BM = biomass.

Condition	PDO	Eth.	But.	Buty.	Acet.	Form.	Suc.	Lac.	BM	H_2	CO_2
R525	16.7%	8.0%	51.3%	2.2%	0.8%	0.3%	0.4%	0.0%	9.0%	11.3%	-
dhaB	0.0%	13.9%	57.4%	0.0%	1.2%	1.0%	0.0%	0.1%	12.5%	13.9%	-
R525 BES	13.6%	11.2%	50.7%	0.1%	0.9%	0.3%	1.6%	2.0%	8.9%	10.6%	-
dhaB BES	0.0%	19.8%	51.6%	1.2%	1.7%	1.2%	0.6%	1.8%	7.5%	14.6%	-
R525	14.7%	6.3%	40.1%	2.1%	1.0%	0.7%	0.5%	0.0%	10.6%	-	24.1%
dhaB	0.0%	10.9%	45.0%	0.0%	1.4%	2.3%	0.0%	0.1%	14.6%	-	25.7%
R525 BES	11.8%	8.6%	39.0%	0.1%	1.0%	0.8%	2.1%	2.3%	10.3%	-	24.0%
dhaB BES	0.0%	15.4%	40.2%	1.1%	2.0%	2.7%	0.8%	2.1%	8.8%	-	26.9%
